# Germline mutation in the *TP53* gene in uveal melanoma

**DOI:** 10.1038/s41598-018-26040-0

**Published:** 2018-05-16

**Authors:** Nikola Hajkova, Jan Hojny, Kristyna Nemejcova, Pavel Dundr, Jan Ulrych, Katerina Jirsova, Johana Glezgova, Ivana Ticha

**Affiliations:** 1 Institute of Pathology, First Faculty of Medicine, Charles University and General University Hospital in Prague, Prague, Czech Republic; 2 1st Department of Surgery – Department of Abdominal, Thoracic Surgery and Traumatology, First Faculty of Medicine, Charles University and General University Hospital in Prague, Prague, Czech Republic; 3 Clinic of Pediatrics and Adolescent Medicine, First Faculty of Medicine, Charles University and General University Hospital in Prague, Prague, Czech Republic; 4 Department of Ophthalmology, First Faculty of Medicine, Charles University and General University Hospital in Prague, Prague, Czech Republic

## Abstract

We performed comprehensive molecular analysis of five cases of metastasizing uveal malignant melanoma (UM) (fresh-frozen samples) with an NGS panel of 73 genes. A likely pathogenic germline *TP53* mutation c.760A > G (p.I254V) was found in two tumor samples and matched nontumor tissue. In three cases, pathogenic *BAP1* mutation was detected together with germline missense variants of uncertain significance in *ATM*. All cases carried recurrent activating *GNAQ* or *GNA11* mutation. Moreover, we analyzed samples from another 16 patients with primary UM by direct Sanger sequencing focusing only on *TP53* coding region. No other germline *TP53* mutation was detected in these samples. Germline *TP53* mutation, usually associated with Li-Fraumeni syndrome, is a rare event in UM. To the best of our knowledge, only one family with germline *TP53* mutation has previously been described. In our study, we detected *TP53* mutation in two patients without known family relationship. The identification of germline aberrations in *TP53* or *BAP1* is important to identify patients with Li-Fraumeni syndrome or *BAP1* cancer syndrome, which is also crucial for proper genetic counseling.

## Introduction

Uveal melanoma (UM) is the most common primary intraocular tumor in adults and represents 3–5% of all melanomas^1,2^. The median age of diagnosis is approximately 62 years. The large majority of ocular melanoma originates from the choroid (95%), ciliary body (5%), and iris (5%)^3^. Over the past decades, the incidence rate has been stable in Central Europe with two cases per million inhabitants. However, this rate increases with latitude^4,5^. Up to half of all patients develop metastatic disease^4,6^. The most common metastatic site is liver (80%-90%), lung (24%), and bone (16%)^2^. Somatic and hereditary mutations of several genes have been described in patients with UM^2,7–11^. However, familial UM is rare and represents less than 1% of all UM, the majority of the families carry germline mutations in the *BAP1* gene^12^.

In our study, we analyzed target region of 73 genes or gene parts by NGS approach in five patients with metastasizing uveal melanoma in order to describe the mutation spectrum and frequency of the variants. Moreover, we analyzed another 16 patients with UM by direct Sanger sequencing focusing only on germline alterations in the *TP53* gene.

## Results

### Spectrum and frequency of genetic variants in mUMs

Nonsynonymous variants in the coding parts of genes and adjacent intronic sequences with variant allele fraction (VAF) ≥10% were evaluated. Genes with clinically relevant variants are listed in Table 1. All variants detected by NGS in five metastatic uveal melanomas (mUM) and nontumor tissue can be found in Supplementary Table S1. Recurrent activating somatic mutation in *GNA11* or *GNAQ* (NM_002067.2: c.626A > T, p.Q209L or NM_002072.3: c.626A > C, p.Q209P, respectively), a typical molecular sign of uveal melanoma, have been detected in all five cases.

**Table 1:** Patients’ and tumors’ characteristics and exonic variants detected in metastatic uveal melanomas by NGS.

No. mUM^a^	age/sex^b^ MFS/OS^c^	Histology; Stage; ciliary body involvement	family history of cancer^d^; other personal anamnesis (age)	Gene (exon)	Mutation cDNA^e^ chromosomal position^f^	Predicted mutation effect	Type^g^	Non-tumor tissue (NGS - VAF %)^h,i^	Liver meta (NGS - VAF %) ^h^	Primary tumor (SQ)^i^	*In silico* prediction ^j^	References^k^
1	54/M 33/98	Mixed; NA; NA	Father †60 Brain Ca, mother †54 Breast Ca;	TP53 (7)	NM_001126112.2: c.760A > G chr17:7577521	p.(I254V)	G	49.7	57.78	NA	Pathogenic	rs746601313
			—	GNA11 (5)	NM_002067.2: c.626A > T chr19:3118942	p.(Q209L)	S	0	27.34	NA	Pathogenic	rs1057519742
2	61/F 24/41	Epithelioid; pT2a; no	NA;	ATM (40)	NM_000051.3: c.5975A > C chr11:108183194	p.(K1992T)	G	47.87	46.09	NA	Pathogenic	rs150757822
			Sclerosis multiplex	BARD 1 (10)	NM_000465.2: c.1973G > A chr:215595163	p.(R658H)	G	53.36	46.73	NA	Neutral	rs377227840
				MSH2 (1)	NM_000251.2: c.4G > A chr2:47630334	p.(A2T)	G	47.15	45.98	NA	Pathogenic	rs63750466
				TJP1 (7)	NM_003257.3: c.704C > A chr15:30053962	p.(T235N)	G	42.57	49.66	NA	Pathogenic	once in GnomAD
				BAP1 (3)	NM_004656.2:c.79delG chr3:52443613	p.(V27Cfs*45)	S	0	29.21	yes	Pathogenic	^46^
				GNA11 (5)	NM_002067.2: c.626A > T chr19:3118942	p.(Q209L)	S	0	24.82	yes	Pathogenic	rs1057519742
3	39/F 28/45	Spindle; NA; NA	NA;	TP53 (7)	NM_001126112.2: c.760A > G chr17:7577521	p.(I254V)	G	51.4	48.19	NA	Pathogenic	rs746601313
			—	CDH1 (16)	NM_004360.3: c.2465C > G chr16:68867218	p.(P822R)	G	41.9	25.65	NA	Pathogenic	novel
				GNAQ (5)	NM_002072.3: c.626A > C chr9:80409488	p.(Q209P)	S	0	33.42	NA	Pathogenic	rs121913492
				SF3B1 (14)	NM_012433.2: c.1874G > T chr2:198267483	p.(R625L)	S	0	32.03	NA	Pathogenic	COSM110695
4	66/F 48/*	Mixed: periphery- epithelioid,	NA;	PARD3 (7)	NM_019619.3: c.875G > C chr10:34688273	p.(S292T)	G	42.7	47.57	NA	Pathogenic	rs62625032
		Central - spindle A, B; pT4a; no	Breast Ca (70)	ATM (37)	NM_000051.3: c.5558A > T chr11:108175463	p.(D1853V)	G	50.51	60.29	NA	Pathogenic	rs1801673
				ATM (48)	NM_000051.3: c.7010_7065dup chr11:108198405–108198462	p.?	G	yes^SQ^	yes	NA	? in-frame	novel
				BAP1 (1)	NM_004656.2: c.2T > A chr3:52443893	p.(M1K)	S	0	60.78	NA	Pathogenic	novel
				GNAQ (5)	NM_002072.3: c.626A > C chr9:80409488	p.(Q209P)	S	0	31.02	NA	Pathogenic	rs121913492
5	54/M 39/*	Spindle B; pT4a; no	—	MLH3 (2)	NM_001040108.1: c.1258G > A chr14:75515101	p.(V420I)	G	yes^SQ^	36.81	yes	Neutral	rs28756982
				ATM (10)	NM_000051.3: c.1522C > T chr11:108121714	p.(L508F)	G	yes^SQ^	50.82	NA	Neutral	once in FLOSSIES
				MET (14)	NM_001127500.1: c.3029C > T chr7:116411990	p.(T1010I)	G	yes^SQ^	49.27	NA	Pathogenic	rs56391007
				SNAI3 (1)	NM_178310.3: c.5C > T chr16:88752810	p.(P2L)	NA^**^	NA	94.04	NA	Pathogenic	rs778704462
				BAP1 (7)	NM_004656.2: c.505_506insC chr3:52441265	p.(H169Pfs*13)	S	no^SQ^	77.59	yes	Pathogenic	novel
				GNA11 (5)	NM_002067.2: c.626A > T chr19:3118942	p.(Q209L)	S	no^SQ^	27.59	yes	Pathogenic	rs1057519742

^a^mUM – metastatic uveal melanoma;

^b^age of primary diagnoses; M – male, F – female; ^c^MFS – liver metastasis free survival (months from primary diagnosis to liver’s metastasis resection or biopsy); OS – overall survival (months from primary diagnosis to death);

*alive;

^d^NA – insignificant or unknown;

^e^GenBank reference sequence + 1 corresponds to the A of the ATG translation initiation codon.

^**f**^According to reference genome GRCh37 (hg19),

^g^G – germline; S – somatic;

^**^Sanger sequencing of exon 1 in *SNAI3* was not performed;

^h^VAF – variant allele fraction, NA – not analyzed/tissue not available, NGS – sequence capture next generation sequencing;

^i^SQ – direct Sanger sequencing;

^j^as pathogenic are considered frameshift variants or variants predicted deleterious in at least 7 out of 13 used predictive programs,

^k^dbSNPdatabase or otherwise specified.

### Patients with germline variants in *TP53*

In two of five patients with mUMs, identical germline *TP53* mutation NM_001126112.2: c.760A > G, p.I254V was detected (representative visualization in IGV and confirmation by Sanger sequencing is in Supplementary Fig. S1). These two patients are unrelated according to family history, and the probability of kinship was furthermore excluded by a bioinformatics approach. One of the carriers was a male patient with primary diagnosis of uveal melanoma at the age of 54, his father had a brain tumor at 60 and his mother had breast cancer at 54. Both parents were not genetically tested and archive tissue for retrospective mutation analysis of *TP53* is unavailable. The second carrier was a female patient diagnosed with uveal melanoma at the age of 39, a family history of cancer has not been demonstrated. Immunohistochemical (IHC) analysis performed on liver tissue slides showed wild-type expression of the p53 protein.

To screen germline *TP53* mutations in a larger cohort, the *TP53* mutation analysis by direct Sanger sequencing was performed in additional 16 nontumor tissue from unrelated patients with UM. No other patient with *TP53* germline mutation was found.

Both mUM samples with germline *TP53* mutation did not have a deletion of chromosome 3 and carried a duplication of 8q (Supplementary Fig. S2 and Supplementary Table S2). One of them also carried a somatic mutation in *SF3B1* (NM_012433.2: c.1874G > T, p.R625L). Further, one of these patients carried a germline mutation in *MSH2* (NM_000251.2: c.4G > A, p.A2T). This variant is probably damaging according to the *in silico* prediction programs, but IHC examination showed intact nuclear expression of all MMR proteins (MSH2, MSH6, MLH1, PMS2) and fragment analysis showed microsatellite stable phenotype.

### Patients with somatic mutations in *BAP1* and germline variants in *ATM*

A germline mutation of *ATM* and a somatic mutation in *BAP1* were found in three patients. None of these patients have a mutation of *TP53*. One patient carried a mutation in *ATM* (NM_000051.3:c.5975A > C, p.K1992T) and had a somatic frameshift mutation in *BAP1* (NM_004656.2:c.79delG, p.V27Cfs*45, VAF 29.21%). Other patient had a somatic mutation in the *BAP1* first coding amino acid that leads to the change of methionine to lysine (c.2T > A, p.?, VAF 60.78%). CNV analyses suggested a partial deletion of chromosome 3, and the patient carried a germline missense mutation in *ATM* (c.5558A > T, p.D1853V) and a germline tandem duplication of 56 bp in exon 48 of the *ATM* gene (c.7010_7065dup56, p.?) (Supplementary Fig. S3). Exon 48 of the *ATM* gene is a part of the regulatory FAT domain that inhibits ATM kinase activity until the occurrence of DNA damage^13^. This patient developed uveal melanoma relatively late at age 66, and breast cancer was diagnosed at 70.

The third patient without *TP53* mutation carried germline mutation in the *ATM* gene (c.1522C > T, p.L508F), and somatic mutation in *BAP1* (c.505_506insC, p.H169Pfs*13, VAF 77.59%) and CNV analyses suggested a partial deletion of chromosome 3.

## Discussion

The Cancer Genome Atlas (TCGA) has recently published comprehensive data of 80 uveal melanomas and suggested four prognostic subtypes^11^. The most prevalent somatic mutations were *GNAQ* (guanine nucleotide-binding protein G(q) subunit α) (50%), *GNA11* (guanine nucleotide-binding protein subunit α-11) (45%), *BAP1* (BRCA1 associated protein-1 (ubiquitin carboxy-terminal hydrolase) (32.5%), *SF3B1* (splicing factor 3b, subunit 1) (22.5%), and *EIF1AX* (Eukaryotic Translation Initiation Factor 1 A, X-Linked) (12.5%). Mutant *GNAQ* was shown to activate the MAPK pathway and it may also have important effects on other pathways such as the phosphatidylinositol-calcium second messenger system^2^. Hot-spot mutations affecting amino acid Glutamine at codon 209 in *GNAQ* or its paralog *GNA11* are mutually exclusive, they have also been detected in benign uveal nevi and are not sufficient for full malignant transformation to melanoma^2,10^. Total loss of function of *BAP1*, that codes the protein involved in DNA damage control, correlates with increased metastatic potential. Partial or complete monosomy of chromosome 3, where *BAP1* (3p21.31-p21.2) is located, is a relatively common event in metastasizing uveal melanoma. Other common chromosomal changes include gain or loss of 1p, 6q, 8q, 8p and less frequently 9p, and 16q. There is a myriad of combinations of such cytogenetic changes. Monosomy of chromosome 3, gain of 8q, epithelioid–mixed cell type and/or larger tumor diameter were strongly associated with a poor prognosis and potential to metastasize^2,14,15^.

In our study, comprehensive molecular analysis of five cases of mUMs was performed. We detected germline *TP53* mutation in 2/5 patients and germline *ATM* mutation together with somatic *BAP1* alteration in another 3/5 patients. Somatic *GNAQ*/*GNA11* recurrent mutation was detected in all 5 cases. Further, somatic or germline variants were detected in *BARD1*, *CDH1*, *MET*, MMR genes, *PARD3*, *SF3B1*, *SNAI3* (Table 1). Based on the unexpected finding of germline *TP53* mutation, we analyzed another 16 patients with UM focusing only on germline *TP53* mutations. However, no other *TP53* mutation was found.

The *TP53* gene is highly polymorphic in coding and noncoding regions and some of these polymorphisms have been shown to increase cancer susceptibility^16^. The frequency of de novo *TP53* germline mutation has been estimated up to 30%, which is very high compared with the frequency of mutations in other tumor suppressor genes^17,18^. Germline mutations in *TP53* are linked to Li-Fraumeni syndrome (LFS). Germline missense mutations are the most common *TP53* variants, occurring in approximately 70% of cases and mainly altering residues within the DNA-binding domain^19^. Patients with LFS are predisposed to a wide variety of cancer types, with early onset, and with the potential for multiple primary cancer sites, including breast cancer, brain tumor, soft tissues cancer, adrenocortical carcinoma and other types^16^. Association of cutaneous malignant melanoma with LFS is relatively rare^20–22^. Moreover, a case of mucosal melanoma has been associated with LFS^23^.

Somatic mutations in the *TP53* gene are one of the most frequent alterations in human cancers. Somatic *TP53* mutations were detected in 12–19% cases of cutaneous malignant melanoma^24–26^ and also described in melanocytic tumor originating in the central nervous system^27^. In uveal melanoma, the occurrence of a somatic mutation is rare^28–33^. However, most researchers use immunostaining with an antibody against p53 protein to detect aberrant protein expression in UM instead of mutation analysis by sequencing of the *TP53* gene. According to the dataset from IARC TP53 database, detected missense mutation show positive IHC results in 88% of cases. Not only *TP53* mutations but also a disturbed p53 pathway can result in abnormal p53 expression^34^.

On the contrary, germline *TP53* mutations in UM is, according to the literature, exceedingly rare and has been described only once in a British family with four generations burdened with uveal melanoma^35^. Here, we describe two other unrelated Czech patients with uveal melanoma that are carriers of germline *TP53* p.I254V mutation. However, pathogenicity of the *TP53* variant p.I254V is not fully elucidated. Foretova *et al*. described a Czech family with LFS (family without evidence of uveal melanoma) and causal *TP53* mutation p.I254V^36^. The result of functional analysis of p53 transactivation ability in yeast (FASAY)^37^ has confirmed that p.I254V is a fully inactivating mutation. Codon 254 is buried in DNA binding domain and located in conserved beta-sheet structure according to the 3D model of p53 (Supplementary Fig. S4)^38^. Any change in this conserved domain could potentially lead to change of conformation, even though amino acids Isoleucine and Valine has similar physicochemical properties. On the contrary, *in silico* analyses suggested deleterious effect of this variant due to its impact on protein structure and function. The ClinVar database (https://www.ncbi.nlm.nih.gov/clinvar/, accessed November 2017) contains data from two submitters and indicates uncertain significance of this germline *TP53* variant. Halvorsen *et al*. detected germline variant p.I254V in *TP53* in 3 out of 394 patients with lung cancer and designated this variant as polymorphism. Nevertheless, we need to take into consideration that this opinion is not based on strong arguments^39^. On the other hand, in the same codon 254 another two missense variants p.I254T and p.I254L were described, either somatic or germline in LFS family^40^, that were considered pathogenic throughout the databases and the literature. Somatic mutation p.I254V has been described according to the IARC TP53 database (http://p53.iarc.fr; assessed December 2017) in seven different tumors so far (Table 2).

**Table 2:** Literature review of reported somatic *TP53* mutation p.I254V.

Topography	Morphology	p53 IHC	Reference*
Esophagus, NOS	Adenocarcinoma, NOS	positive	^47^
Mouth, NOS	Squamous cell carcinoma, NOS	positive	^48^
Connective, Subcutaneous and other Soft tissues of abdomen	Hemangiosarcoma	NA	^49^
Breast, NOS	Carcinoma, NOS	positive	^50^
Lymph nodes, NOS	Malignant lymphoma, large B-cell, diffuse, NOS	NA	^51^
Nasal cavity (excludes Nose, NOS)	NK/T-cell lymphoma, nasal and nasal-type	NA	^52^
	Adult acute lymphoblastic leukemia	NA	^53^

*According to IARC TP53 database (assessed November 2017); NA – not analyzed.

Inherited pathogenic *ATM* mutation is the cause of autosomal recessive disease ataxia-telangiectasia which predisposes the individuals for increased cancer risk^41,42^. Germline or somatic mutations in *ATM* have not been described in uveal melanoma so far. In our study, we detected germline *ATM* variants in three patients with metastatic uveal melanoma. Nevertheless, all variants were of unknown significance or likely benign. Three of them have been described previously and their population mutant allele frequency (MAF) is low: p.K1992T, MAF(gnomAD) = 0.0003; p.D1853V, MAF(gnomAD) = 0.004915, and p.L508F, which is only described in database FLOSSIES MAF = 0.0001. In all three of our cases, *ATM* mutations cooccurred with pathogenic somatic variants in the *BAP1* gene and two tumors have partial loss of chromosome 3, which are also relatively common event in metastasizing uveal melanoma, both associated with worse prognosis. Not only somatic but also germline mutation of *BAP1* gene may occur in UM and the majority of familial UM with germline *BAP1* mutation is associated with BAP1-tumor predisposition syndrome, which also increases the risk of the atypical Spitz tumors, malignant mesothelioma, and cutaneous melanoma^43^.

## Conclusion

In conclusion, the results of our study have shown that genetic changes occurring in UM can be very heterogeneous. In three tumors and/or metastases we detected pathogenic somatic mutations in *BAP1*, which is not an unexpected finding, but these mutations occur in all three patients together with the germline missense variant in *ATM*. Despite the fact that detected *ATM* variants are of uncertain significance, this finding deserves further research because *ATM* mutations have never been reported in UM to date. Moreover, we have found germline *TP53* mutation in two patients. Germline *TP53* aberration has been described in UM in only one family so far, but the true incidence of *TP53* mutations in UM is difficult to estimate due to the sparse studies focusing on this topic. However, the identification of germline *TP53* or *BAP1* mutations is important to be able to identify patients with Li-Fraumeni syndrome or BAP1 cancer syndrome and is the first step for proper genetic counseling and management of the patients and family members. We are well aware of the limitations of our study, which are mainly due to the small sample set. Nevertheless, we believe that the results of our study broaden the knowledge of molecular changes occurring in such a rare tumor as UM.

## Material and Methods

### Patients and samples

Archive files of the Bank of Biological Material of the First Faculty of Medicine, Charles University, Prague, were searched for uveal malignant melanomas or their metastasis. Five fresh frozen liver metastasis (stored in liquid nitrogen at −180 °C) of uveal melanoma (mUM) from 5 patients were found. For all 5 mUM cases, formalin-fixed paraffin-embedded tissue (FFPE) blocks of liver metastasis were also available for subsequent IHC analysis. Corresponding fresh-frozen nontumor tissue was available in 4/5 patients and genomic DNA from blood was available in 1/5 patient. Characteristics of the patients and tumors are summarized in Table 1. For 2/5 patients, FFPE blocks of matching primary uveal tumors were available in the archive files of the Institute of Pathology, First Faculty of Medicine, Charles University and General University Hospital in Prague. For extended analysis of *TP53* mutation status in UM, the same archive files (between 2007 and 2017) were searched and another 16 patients (mean age 50, range 20–79 years) with available FFPE tissue were found. Review of the hematoxylin and eosin stained slides was performed in all cases and areas of nontumor or tumor tissues for macrodissection were marked (with estimation of tumor cell percentage in the selected area; ranges between 60–99%).

### Ethics Statement

In compliance with the Helsinki Declaration, the study has been approved by The Ethics Committee of General University Hospital in Prague and an informed consent document was signed by participants. The methods were carried out according to the approved ethical guidelines.

### DNA isolation

DNA from fresh-frozen tissue or FFPE blocks was isolated using standard procedures implementing QIAamp DNA Tissue kit (Qiagen) or cobas® DNA Sample Preparation kit (Roche; Germany), respectively.

### NGS

Whole project and all auxiliary files are designed for genome build GRCh37 (hg19) coordinates. Samples for sequence capture NGS (massive parallel sequencing) were prepared using the KAPA HyperPlus kit. Target sequences were enriched using commercial hybridization probes (Nimblegen, Roche) designed to human DNA regions of our interest (Supplementary Table S3; 219 kbp). Library was pair-end sequenced by MiSeq instrument (Illumina). Processing of raw sequencing data was performed to analyze spectrum of genetic variants, such as single nucleotide variants and short insertions or deletions, and copy number variations (CNVs) using NextGENe software (Softgenetics, State College, PA) according to standardized biostatistical methods for NGS data (detailed setting in Supplementary Methods). Nonsynonymous variants in exons and adjacent intronic regions with minimal average coverage 100x and frequency ≥10% were evaluated and manually controlled using IGV viewer (Broad Institute). The data of average coverage of each region can be found as Supplementary Table S4.

### Sanger sequencing

Detected variants with a frequency higher than 10% were confirmed by direct Sanger sequencing using BigDye v3.1 and ABI3500 analyzer (ThermoFisher). Selected variants were also checked in primary tumors from respective cases, available for 2/5 cases. Nontumor tissue or genomic DNA from blood was used to determine germline or somatic state of detected variants.

Further, direct sequencing of the *TP53* coding- and adjacent intronic sequences (NM_001126112.2: exons 2–11) was performed in 16 primary uveal melanomas. Primers used for amplification and direct Sanger sequencing are available in Supplementary Table S5.

### Microsatellite instability

Analysis of microsatellite instability (MSI) was performed with the set of five quasimonomorphic mononucleotide microsatellite markers BAT-26, BAT-25, NR-21, NR-22, NR-24. Fragmentation analysis was performed on ABI 3500 (ThermoFisher). MSI-high or MSI-low phenotype were defined as the presence of two or more- or one instable loci, respectively. MSI stable tumors (MSS) show no instability.

### Immunohistochemical analysis

Immunohistochemical analysis (IHC) was performed using the avidin-biotin complex method with an antibody against p53 (BP 53–12, dilution 1:200, Zytomed Systems, Berlin, Germany). Additional IHC in the case mUM_2 (carrier of the mutation in MSH2 c.4G > A, p.A2T) was performed with antibodies against mismatch repair (MMR) proteins including MSH2 (clone FE 11, dilution 1:50, Zytomed Systems), MSH6 (clone 44, dilution 1:50, Zytomed Systems), MLH1 (clone G168-15, dilution 1:200, Spring Bioscience, Pleasanton, CA), PMS2 (EPR3947, ready-to-use, Zytomed Systems). Antigen retrieval was performed in 0.01 M citrate buffer (pH 6.0) for 40 minutes in a water bath at 98 °C for p53; in Dako Target Retrieval Solution (pH 9.0) overnight at 98 °C for MSH2, MSH6, MLH1 and for 50 minutes at 98 °C for PMS2.

### Biostatistical analysis

NextGENe® Software was used for the analysis of the sequencing data, and CNV analysis. The Pindel tool was used to detect break points of large deletions and medium sized insertions^44^. The R package SNPRelate was performed to exclude unknown family relationships^45^.

### Copy number variation

Used custom NGS panel was not primarily designed for analysis of copy number variation (CNV), so only regions with sufficient coverage by target genes were analyzed. Each region of our custom panel and their chromosomal location are listed in Supplementary Table S6. CNV analysis was performed focused on chromosome 3, 6, 8, 16, according to the current knowledge about cytogenetic changes in uveal melanoma. CNV tool Dispersion and Hidden Markov Model (HMM; part of NextGENe Software) was used to evaluate CNV variations between tumor tissue and corresponding nontumor tissue (mUM_1, mUM_2, mUM_3, mUM_4) and in case mUM_5 for comparison were used normalized coverage of the pool of other 4 nontumor samples.

### *In silico* prediction tools

In order to assess the impact of detected missense variants, we employed several widely used *in silico* prediction programs or databases, which are imported in NextGENe Software.

Clinical significance and ensemble prediction scores from the ClinVar database, COSMIC database, and dbNSFP database (MetaSVM, MetaLR, RS_DBSNP141, SIFT, Polyphen2_HDIV, Polyphen2_HVAR, LRT, MutationTaster, MutationAssessor, FATHMM, PROVEAN GERP++, phyloP46, SiPhy) were imported to NextGENe mutation report (Supplementary Table S1).

Mutant allele frequencies for ATM aberrations were searched in databases The Genome Aggregation Database (gnomAD) and Fabulous Ladies Over Seventy database (FLOSSIES).

### Data availability

All data generated or analysed during this study are included in this published article (and its Supplementary Files).

## Electronic supplementary material

Suppplementary Methods and Figures

Mutation report of all detected variant processed by Nextgene

CNV analysis processed by Nextgene

Target area in custom NGS panel

Average coverage of all regions

Primers used for Sanger sequencing analysis

Regions covered in CNV analysis
