# Supplementary material for: Germline mutation in the TP53 gene in uveal melanoma
Source: Sci Rep. 2018 May 16;8:7618. doi: 10.1038/s41598-018-26040-0 (PMC5955881; doi:10.1038/s41598-018-26040-0)
Supplement: Supplementary file 1 — Suppplementary Methods and Figures [file 41598_2018_26040_MOESM1_ESM.pdf]

Supplementary Methods and Figures

Germline mutation in the *TP53* gene in uveal melanoma

Nikola Hajkova, Jan Hojny, Kristyna Nemejcova, Pavel Dundr, Jan Ulrych, Katerina Jirsova, Johana Glezgova, Ivana Ticha

Supplementary Methods: Pipeline for processing of raw NGS data

Primary raw data were trimmed and demultiplexed by MiSeq system during post-sequencing process. Output in .fastq format was complexly analyzed by NextGENe software (Softgenetics).

The PCR duplicate reads were removed by Sequence Operation Tool (default settings), then .fastq files were converted by using Format Conversion Tool to .fasta format. During conversion, reads with low quality were removed (Settings: Median score threshold ≥ 25; Max # of uncalled bases ≤ 2; Called base number of each read ≥ 40; Trim or reject read when ≥ 3 base(s) with score ≤ 2).

After format conversion, reads were mapped on genome by Project Wizzard Tool (Settings: Instrument type – Illumina; Application type – SNP/Indel discovery; Steps – Sequence Alignment; Reference file – Human_GRCh_v37p10_dbsnp135; Allowable mismatched bases – 0; Allowable ambiguous alignments – 10; Seeds: 21 bases, move step – 1 base; Allowable alignments – 80; Overall matching base percentage ≥ 95%). Results of NextGENe software analysis (mutation report, expression report, coverage report, CNV analysis report) were filtered for the region of interest and mutation report additionally for the frequency of mutation allele >5%.

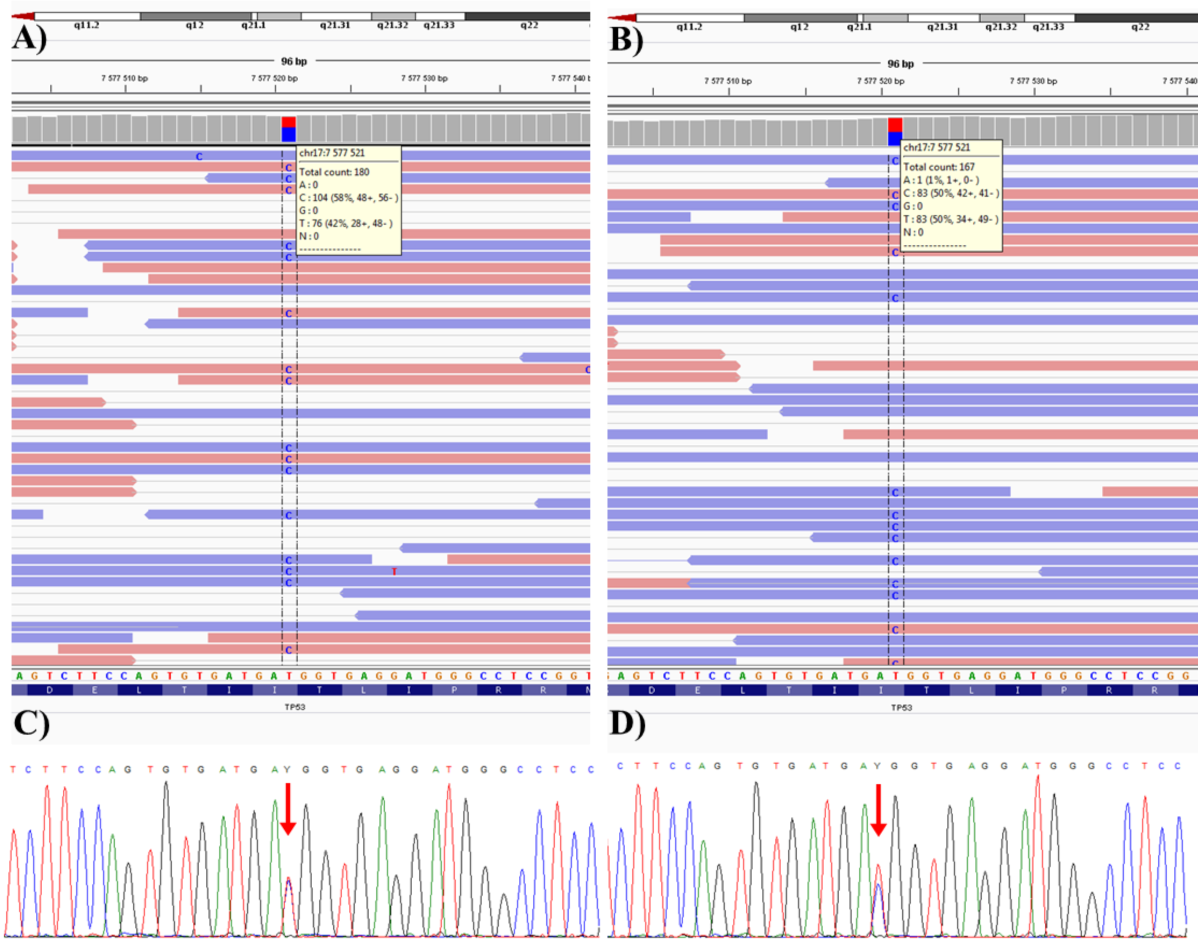

Supplementary Figure S1: IGV visualization of germline mutation p.I254V in *TP53* in sample 1 (A) and corresponding non-tumor tissue (B), and corresponding electropherograms of Sanger sequencing analysis confirming *TP53* mutation p.I254V in sample 1 (C) and corresponding nontumor tissue (D). Red arrows are indicating missense mutation.

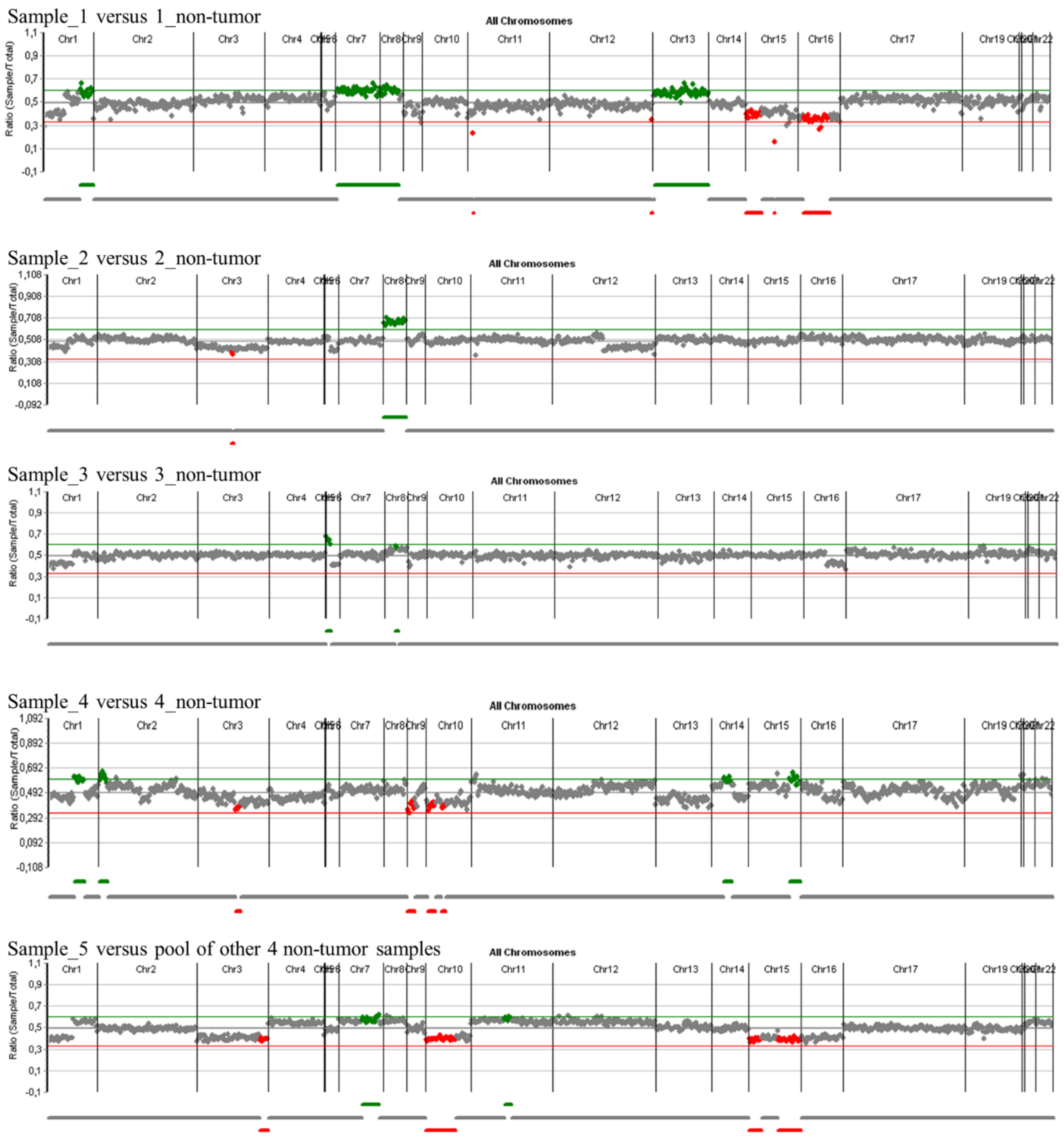

Supplementary Figure S2: Visualization of CNV analysis of metastatic uveal melanomas – by CNV tool Dispersion and Hidden Markov Model (NextGENe Software), Corresponding raw data are in Supplementary Table 2.

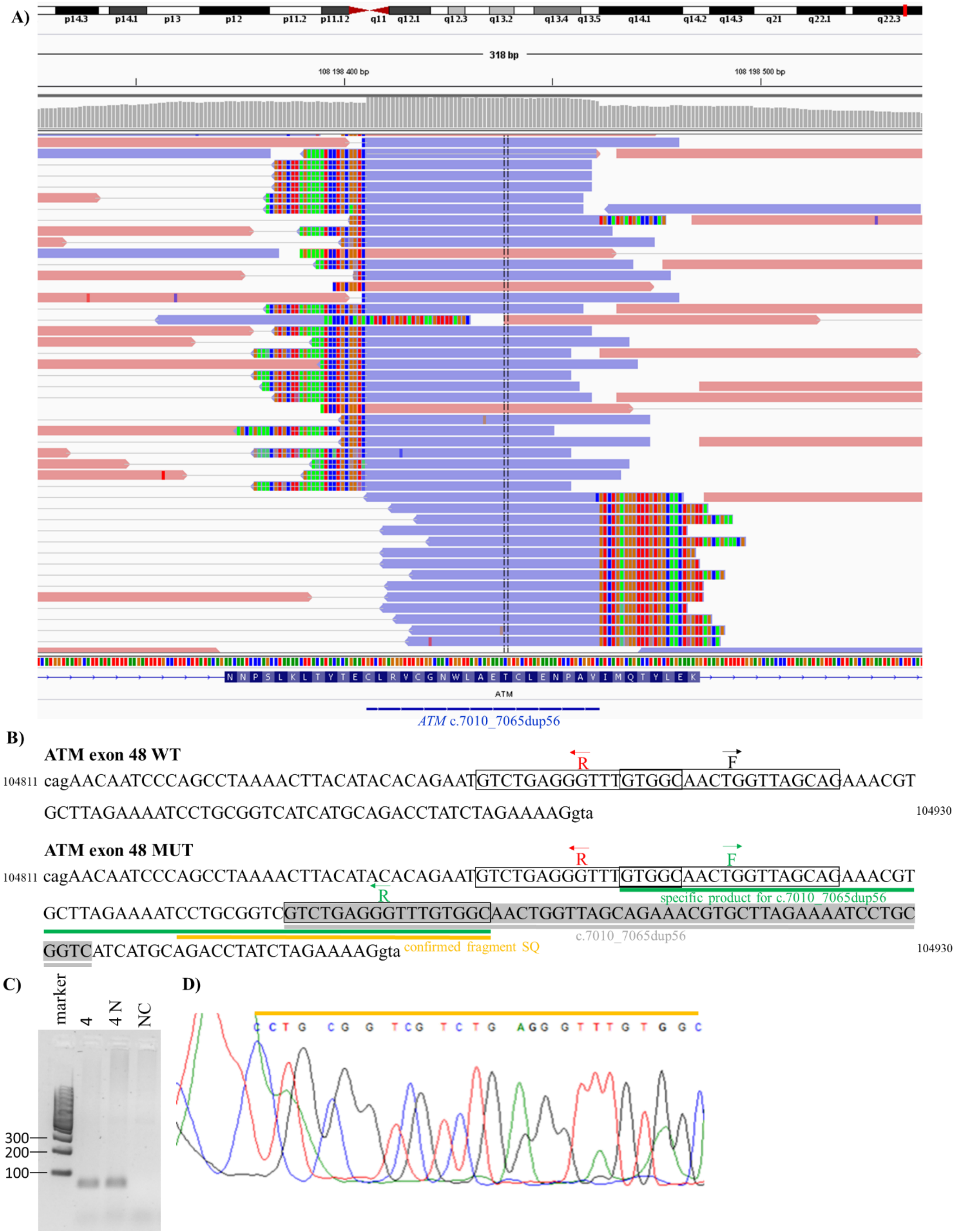

Supplementary Figure S3: Germline tandem duplication in *ATM* in exon 48 NM_000051.3: c.7010_7065dup56 in sample 4. **A)** Visualization of data processed by „Pindel tool" in IGV viewer (Broad Institute). **B)** Specific primers used for PCR and Sanger sequencing are marked in the box. Forward primer (5'-GTGGCAACTGGTTAGCAG-3') was located several bases before duplication and reverse primer (5'-GCCACAAACCCTCAGAC-3') was located at the

beginning of duplicated region to amplify specific product (green underline) that includes duplication (highlighted in grey). **C)** Visualization of PCR specific product (71bp) that includes *ATM* duplicated region (56bp). **4** – tumor tissue of metastatic uveal melanoma sample 4, **4 N** – nontumor tissue of sample 4, **NC** negative control. **D)** confirmatory electrophoreogram of a fragment that includes tandem duplication, sequenced with forward primer.

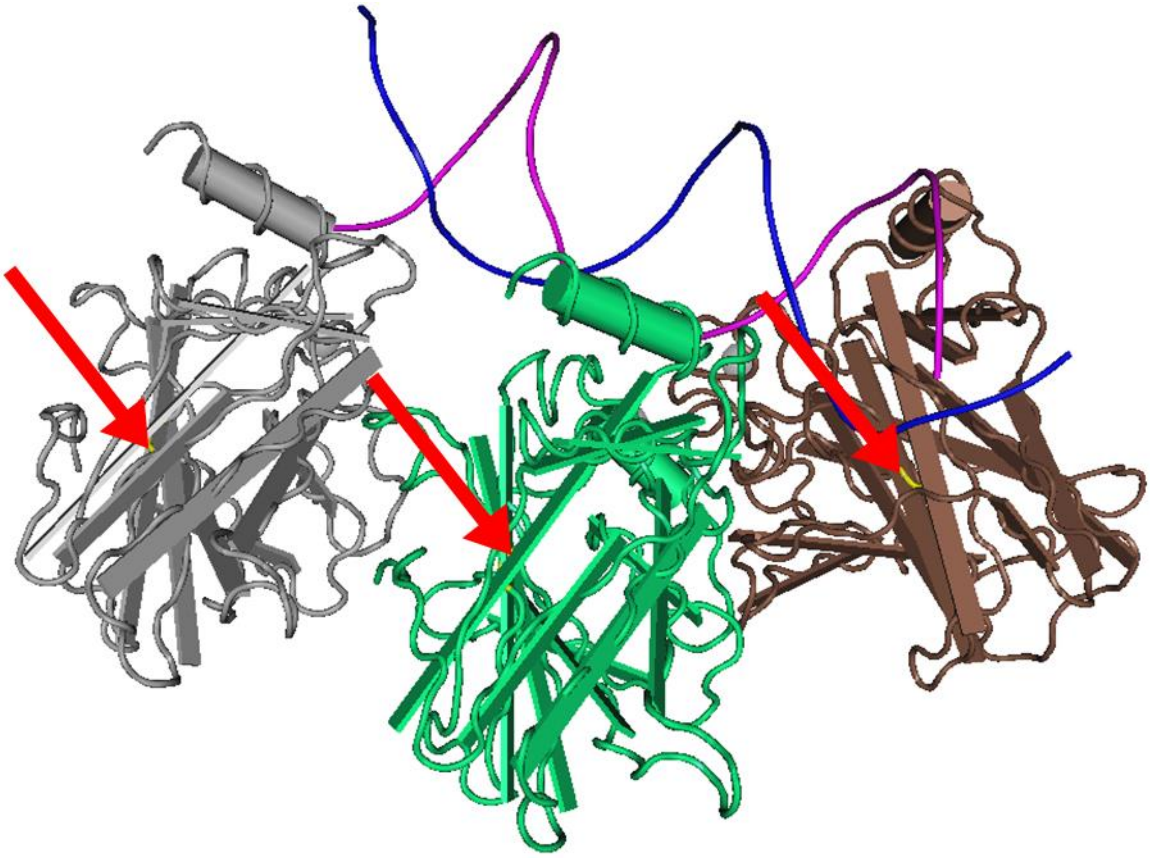

Supplementary Figure S4: 3D structure of p53 complex with DNA using Cn3D 4.3.1 3-D structure viewer (https://www.ncbi.nlm.nih.gov/Structure/CN3D/cn3dinstall.shtml) to show the location of codon 254.

The codon 254 (yellow or red arrows indicates the position) is located in beta-sheets, buried in DNA binding domain. The 3D model contains three p53 core domain molecules and one DNA strand (blue and purple). Two of the core domains bind DNA; one (green) interacts extensively with a consensus binding site, and the other (grey) binds at a nonconsensus site at the interface of DNA fragments related by crystallographic symmetry. The third core domain molecule (brown) does not bind DNA but makes protein-protein contacts stabilizing crystal packing. The zinc atoms are shown as grey spheres.
